# Detecting emotions through EEG signals based on modified convolutional fuzzy neural network

**DOI:** 10.1038/s41598-024-60977-9

**Published:** 2024-05-06

**Authors:** Nasim Ahmadzadeh Nobari Azar, Nadire Cavus, Parvaneh Esmaili, Boran Sekeroglu, Süleyman Aşır

**Affiliations:** 1 Department of Biomedical Engineering, Near East University, 99138 Nicosia, Cyprus; 2 Department of Computer Information Systems, Near East University, 99138 Nicosia, Cyprus; 3 Computer Information Systems Research and Technology Center, Near East University, Nicosia, 99138 Turkey; 4 Department of Computer Engineering, Cyprus International University, 99258 Nicosia, Cyprus; 5 Software Engineering Department, World Peace University, Nicosia, Turkey; 6 Center for Science and Technology and Engineering, Near East University, Nicosia, 99138 Turkey

**Keywords:** Biomedical engineering, Neural decoding

## Abstract

Emotion is a human sense that can influence an individual’s life quality in both positive and negative ways. The ability to distinguish different types of emotion can lead researchers to estimate the current situation of patients or the probability of future disease. Recognizing emotions from images have problems concealing their feeling by modifying their facial expressions. This led researchers to consider Electroencephalography (EEG) signals for more accurate emotion detection. However, the complexity of EEG recordings and data analysis using conventional machine learning algorithms caused inconsistent emotion recognition. Therefore, utilizing hybrid deep learning models and other techniques has become common due to their ability to analyze complicated data and achieve higher performance by integrating diverse features of the models. However, researchers prioritize models with fewer parameters to achieve the highest average accuracy. This study improves the Convolutional Fuzzy Neural Network (CFNN) for emotion recognition using EEG signals to achieve a reliable detection system. Initially, the pre-processing and feature extraction phases are implemented to obtain noiseless and informative data. Then, the CFNN with modified architecture is trained to classify emotions. Several parametric and comparative experiments are performed. The proposed model achieved reliable performance for emotion recognition with an average accuracy of 98.21% and 98.08% for valence (pleasantness) and arousal (intensity), respectively, and outperformed state-of-the-art methods.

## Introduction

In recent years, the study of emotion recognition has become increasingly popular among researchers from diverse backgrounds. This is likely due to the ability of emotion recognition to reveal important aspects of individual behavior and mental states. Affective computing is a relatively new research field that aims to provide computer systems to Affective computing being a relatively new research field is aimed at using computer systems to detect, analyses, and interpret emotional information provided by people effectively^1^. This form of computing enables researchers understand how people feel, what triggers their feelings, and how to design a more similar, responsive, and better systems to meet people's needs. However, one of the most challenging aspects of this technology is in development of computer methods and approaches which aids the natural interaction of computers with humans. This phenomenon is known as human–computer interaction (HCI) as it analyses and evaluate emotional exchanges and emotional state existing between a person and machine interaction^2^.

For a better understanding of emotional states and exchanges, two categorized methods have been proposed. The first method utilizes effective conduct characteristics, such as speech intonation, facial gestures, and body language for the detection of these category of emotion However, the second group considers the signals of physio-logical activities recorded by non-invasive sensors to detect emotions as electrical responses^3^. Emotions have been primarily represented in two ways in the related emotion recognition literature. The primary approach categorizes emotions as distinct states, including the six fundamental emotions suggested by Ekman and Friesen^4^. According to the second approach, emotion is expressed as a continuous 4-D space of valence, arousal, dominance, and liking^5^. However, most investigations reduce this space to 2-D, applying valence and arousal dimensions^6^.

Many researchers have found that the generation and activity of emotions are very closely related to the activity of the brain's cortex^7^, and the EEG (Electroencephalogram) has been recently utilized for monitoring brain activity due to its high detection sensitivity in comparison with other methods^8^. However, the number of channels and various frequency bands in recordings complicate the analysis and require advanced tools. Since machine learning (ML) and deep learning (DL) are effective approaches due to their ability to relate between features and make decisions, they are frequently used for analyzing EEG signals to solve the problem mentioned above^9^. However, ML comprises data preparation, feature selection, and classification steps, which generally require manual procedure and cause loss of relevant data by increasing the computation cost in data preparation^10^. Numerous models, including K-Nearest Neighbor (KNN), Support Vector Machine (SVM), Decision Tree (DT), and Random Forest (RF) encompass a wide spectrum of machine learning techniques. Since the K-Nearest-Neighbors (KNN) technique is nonparametric, it doesn’t make any assumptions about the elementary dataset. Its simplicity and efficacy are well known. It's an algorithm for supervised learning. In order to predict the class of the unlabeled data, a labeled training dataset with data points categorized into many classes is supplied^11^.

Support Vector Machines (SVM) are machine learning techniques that use binary linear classification to divide classes based on how much the instances of their boundary line differences differ from one another. It is called the optimal margin classifier for this reason^12^.

On the contrary, recent advances in deep learning technology have made it very successful at recognizing things such as pictures, speeches, and text. This is because deep learning technology can learn to recognize complex, high-level features on its own, and it requires less time to extract the features of a particular object. The reasonable and superior results achieved by the 1D convolutional neural networks (CNN) made it one of the benchmark algorithms in EEG analysis^10^. Extracting the features in the pre-defined window (periods) of the recordings in the convolutional layer decreased the computational cost spent for the data preparation and reduced the noise dependency of the analysis. However, the similarity of the signals and the channel variety require an approximation of the signals to improve the accuracy of emotion recognition. The deep-bidirectional LSTMs (Bi-LSTM) represent an advancement in conventional LSTM models by incorporating two LSTMs into input data processing. Initially, an LSTM processes the input sequence, followed by another round where the LSTM model processes the input sequence in reverse order. Utilizing the LSTM twice enhances the ability to learn long-term dependencies, thereby enhancing the model's accuracy^13^.

On the other hand, neural networks and fuzzy systems are adequate for the universal approximation for modelling nonlinear functions. Therefore, the fuzzy neural network (FNN) is a hybrid model that merges the capabilities of both neural networks and fuzzy logic into a single, cohesive system^14^. The FNN offers the benefit of improving the efficacy of function estimation.

This paper proposes a hybrid Convolution Fuzzy Neural Network (CFNN) model for emotion classification on the DEAP database^5^. The proposed model aims to provide a more comprehensive and accurate understanding of emotions within the dataset by combining multiple approaches and techniques. In the proposed method, signals are used as inputs of CFNN for the first time, and the method comprises a layer for converting flattened features obtained in the convolutional layer into fuzzy quantities (fuzzification) with a following layer for converting fuzzy sets into crisp values (defuzzification). The suggested approach enhances classification accuracy by utilizing fuzzy neural networks, which have the capability to produce not just precise values but also fuzzy values. This implies that fuzzy sets potentially contain additional information, leading to improved accuracy. Moreover, the model is adept at managing the noise disruption in the data. Therefore, it demonstrated improved capabilities for recognition and classification. Additionally, the study highlighted the significant impact of utilizing the Fast Fourier Transform (FFT) as a feature extraction technique. Applying FFT to the input signals extracted relevant frequency-domain information, enhancing the classification model's discriminative power.

The rest of this paper's material is organized as follows: section “Related works” summarizes the related works in the literature in a timeline by highlighting the problems. Section “Methods” presents the materials and methods considered in this paper. Section “Proposed method” goes into detail about the proposed method. The “Results” section demonstrates the model's results analysis and discussions. Finally, the last section is the “Conclusion” of the proposed work.

## Related works

Several studies have been conducted on emotion recognition, and most of them have been focused on the ML or DL approach in the last decade.

### Machine learning-related works

Numerous machine learning techniques are used to classify EEG signals, including K-Nearest Neighbor (KNN)^15^, Support Vector Machine (SVM)^16^, Decision Tree (DT)^17^, and Random Forest (RF)^18^. Traditional EEG-based emotion detection algorithms primarily concentrate on extracting individual EEG features from different domains.

Ismael et al.^19^ proposed a technique for categorizing EEG data based on a two-stage majority vote. First, bandpass filters were used to reduce noise from the raw EEG data, and afterward, low-pass filters were used to extract rhythm. The rhythms were analyzed based on their fractal dimensions and wavelet-based entropy features, which were evaluated using KNN and performed on the DEAP dataset. In different studies^20^,^21^ researchers applied SVM using multichannel feature fusion and a dimensional model to detect diverse emotions.

Amiri et al.^22^ proposed applying the DWT approach to extract the EEG signal’s properties. They used the DEAP dataset to categorize real-time affective responses using the arousal-valence dimensions model. The two distinct classifiers, SVM and KNN, were used in this study and achieved reasonable accuracy. They concluded that the gamma, as a high-frequency classifier, provided higher accuracy than the other frequencies.

To investigate the effects of the various frequency bands and number of channels on accuracy, Li et al.^23^ divided the DEAP dataset into four frequency bands. Then, as an input characteristic for a KNN classifier, the entropy and energy of each band were computed. The authors concluded that the gamma frequency band exhibited the highest classification accuracy regardless of the valence or arousal dimension. Also, it was shown that the gamma frequency band, as opposed to the low-frequency band, was significant for the emotional state in the valence and arousal dimensions. Additionally, they demonstrated how adding more EEG channels could enhance the categorization precision of emotional states.

Furthermore, scientists applied three distinct methods^24^ to combine data from various channels and the Fusion after deep feature reduction (FaDFR) method, which combines reduced deep time–frequency features from EEG channels with Inception-V3^25^CNN for deep feature extraction and SVM for classification, produced superior results. The results demonstrated 88.6% accuracy on the DEAP dataset and 94.58% accuracy on the SEED dataset^26^.

Moreover, a novel approach was introduced in^27^ for emotion detection using multichannel EEG data. The framework utilized a linear EEG model and an emotion timing model to improve accuracy in emotion classification. Signal framing, Hamming window, and power spectral density were used to extract the features from the signals. They achieved 81.10% and 74.38% accuracy in valence and arousal on the DEAP dataset, respectively.

### Deep learning-related works

As DL techniques progress rapidly, DL modules may eventually replace all or part of the abovementioned systems' components. Several networks have been proposed based on CNN^28^ and Long-Short Term Memory (LSTM) neural networks^29^.

Xiao et al.^30^ proposed an emotion recognition algorithm that relied on a CNN. EEG signals were mapped into 4D spaces, and Differential Entropy features were extracted from them. The next step involved gaining spatial and spectral information by utilizing the CNN from each temporal piece. LSTM was used to investigate the important aspects of various pieces and identify their emotions by assigning different weights to diverse brain regions and frequency bands. The algorithm obtained excellent classification results. The study was implemented on both DEAP and SEED datasets.

Cimtay et al.^31^ attempted end-to-end methods for the classification of emotions by using CNN. In this research, they developed the model by adding more layers to improve the classification performance. A median filter was utilized to eliminate false identifications along an emotional prediction interval, improving classification accuracy. In the^32^, the model comprised a 1D convolution layer that collects electrode correlations throughout the spatial dimension and receives weighted combinations of contextual data on DEAP and SEED datasets to overcome the limitations of nonlinear estimation and effectively extract features from frequency bands. The technique that was developed can effectively extract features from noisy EEG data while managing electrode correlations and temporal dependencies.

In another research carried out^33^, to gain high accuracy and performance in emotion detection, stacked Bi-LSTM was applied to the DEAP database. The model’s efficiency was enhanced by extracting the statistical, wavelet, and Hurst exponent features. The Binary Grey Wolf Optimizer (BGWO) algorithm managed the problem of the complexity and high dimensionality of the dataset, and it caused a diminishment in classifying time and an improvement in the model's effectiveness.

A newly developed deep learning framework^34^, based on subject-independent, comprises two main components. First, an unsupervised LSTM with a channel-attention autoencoder was suggested for obtaining a subject-invariant latent vector subspace for each subject. Second, CNN with an attention framework was de-scribed for carrying out the task of subject-independent emotion recognition derived from the last step. The method was evaluated on the DEAP, SEED, and CHB-MIT^35^ datasets.

Fusion of the models in deep learning was the challenge faced by the researchers^36^. The fused model employed multiple graph convolutional neural networks (GCNNs) to extract features from the graph domain. Additionally, LSTM cells were utilized to capture the evolving relationships between two EEG channels over time and perform temporal feature extraction. Finally, a dense layer was employed to classify emotions based on the extracted features. The results were superior to the state-of-the-art methods on the DEAP dataset, and the authors concluded that an attention weight, which provides weights to the emotional states that arise at particular moments, was computed to identify the input component that has the most impact on the output; the greater the importance of the relevant input component, the higher the magnitude in the network training.

In another study^37^, a hybrid model consisting of a combination of CNN and LSTM was presented to construct a deep-learning system for emotion identification applied to the DEAP and DREAMER^38^ databases. This method applied CNN and channel-wise attention mechanisms to investigate spatial information. Additionally, attention-based convolutional recurrent neural networks (ACRNN) combined extended self-attention with RNNF to investigate temporal information in EEG signals. Iyer et al.^39^ employed differential entropy (DE) to extract different frequencies based on EEG signals, and a hybrid model was produced by combining CNN and LSTM model sub-blocks. They achieved 65% accuracy on the DEAP dataset and 97.16% on the SEED dataset.

A novel fuzzy rule-based categorization system that uses EEG to measure emotional characteristics was presented in^40^. This study’s purpose was to derive rules from EEG data for a fuzzy categorization automatically. The proposed method extracted a set of rules from the EEG data using FCM. Applying the suggested fuzzy emotion classifier and the fuzzy extraction approach yielded a faster calculation time. Because it learns from data and builds rules, FCFCM can be used for every axis of three-dimensional models of human emotion. According to the results, the algorithm's accuracy was higher than that of SVM and fuzzy classification using pre-defined rules, with ratings of 55.77%, 49.62%, and 54%, respectively.

## Methods

This section presents the considered dataset and evaluation metrics. Additionally, CNN and FNN are briefly described before the proposed model.

### Dataset

In this study, we considered a single but state-of-the-art DEAP dataset^5^, which is one of the gold-standard and common datasets for emotion recognition. The DEAP dataset makes it feasible to assess the quality of the extracted features and the proposed models’ performance.

The dataset consists of 32 subjects (16 male and 16 female) who watched 40 videos to stimulate distinct emotions. The Biosemi ActiveTwo device was used to record EEGs from the individuals while they were watching the clip. Following each film, they completed a Likert scale questionnaire ranging from 1 (low) to 9 (high) to record their degree of arousal, valence, dominance, liking, and familiarity. To achieve accurate and reliable results, since there were adequate observations for each subject in the emotion detection approach, one dataset confirmed the validity of the research.

**Figure 1:**
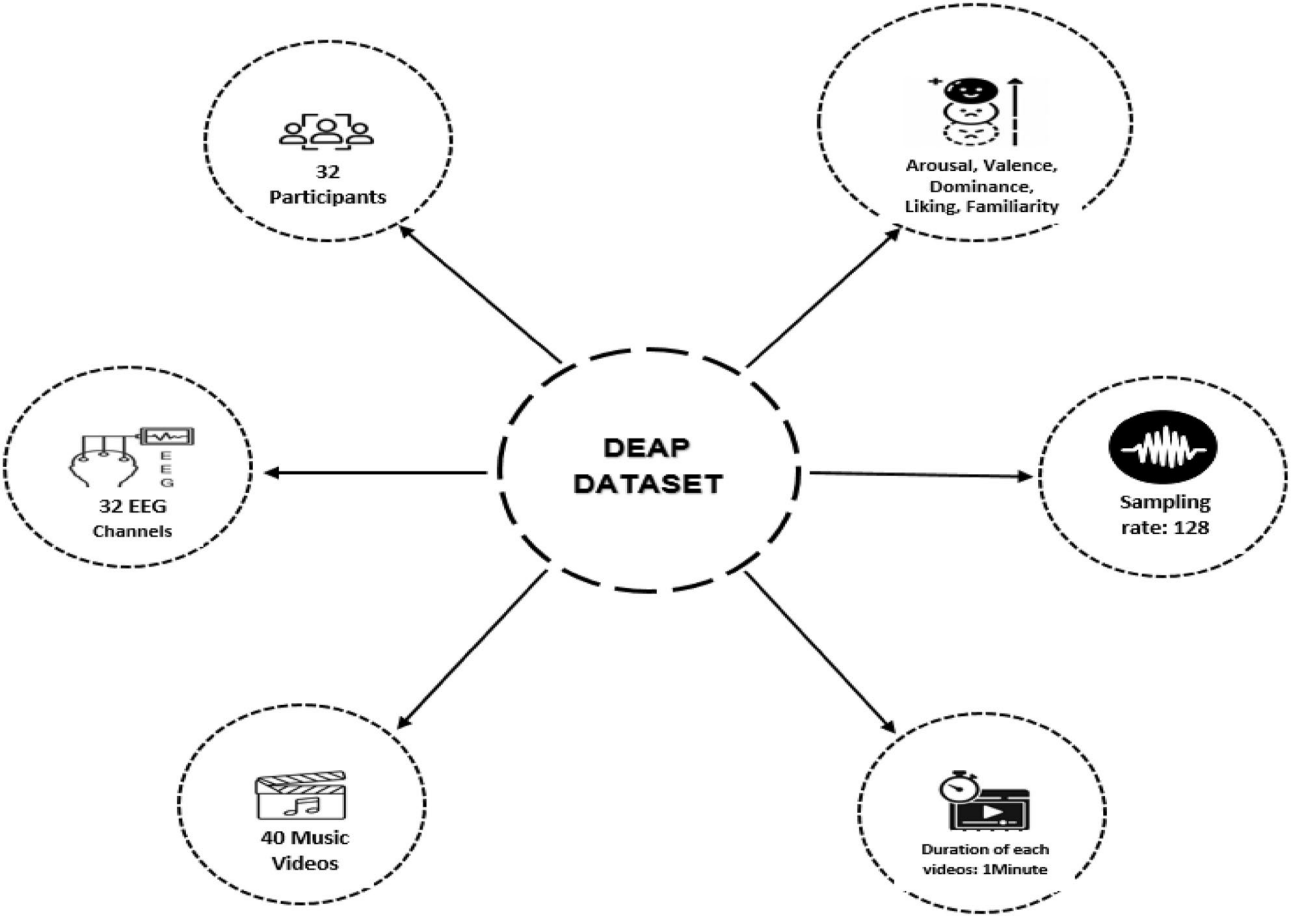
Summary of the DEAP dataset content.

The DEAP dataset is already pre-processed and is accessible to all researchers for use. Music videos are utilized as stimulation for triggering emotions in this dataset. The signals were recorded at 512 Hz with 128 Hz resampling. In this investigation, the employed method involves a 2D emotional model that includes valence and arousal. Valence measures how positive or negative an emotion is, whereas arousal refers to the intensity of related feelings and demonstrates the amount of excitement or apathy. Figure 1 illustrates the overall presentation of the DEAP dataset.

### Evaluation metrics

Several evaluation metrics can evaluate the performance of a model. In our study, we used four common metrics, namely accuracy, precision, recall, and F1-Score, to evaluate and analyze the actual results of the proposed model and to enable comparison with the existing recent studies.

Accuracy is used to measure the general recognition ability of the models. The formula for accuracy is given in Eq. (1).

1 $${\text{Accuracy }} = \frac{{\left( {TP + TN} \right)}}{{\left( {TP + FP + TN + FN} \right)}}$$

where TP, TN, FP, and FN represent True Positive, True Negative, False Positive, and False Negative samples of predicted data.

However, the model's actual performance can be misleading, particularly for imbalanced data and multinomial classification. Therefore, precision and recall help us to analyze how effectively the model recognizes positive or negative samples for each class separately. The formulae of precision and recall are given in Eqs. (2, 3) PRE and REC.

2 $${\text{Precision }} = \frac{{{\text{TP}}}}{{\left( {{\text{TP}} + {\text{FP }}} \right)}}$$

3 $${\text{Recall }} = \frac{TP}{{\left( {TP + FN} \right)}}.$$

The F1-score is an effective evaluation metric, particularly for imbalanced datasets, in order to determine actual performance and the recognition ability of the models. It reduces the effect of the class majority on the results and provides a more balanced score than the accuracy. The formula of F1-Score is given in Eq. (4).

4 $${\text{F1 Score }} = \frac{{\left( {2 \times Precision \times Recall} \right) }}{{\left( {Recall + Precision} \right)}}$$

### Convolutional neural network

CNN plays a significant role in the field of deep learning. It is one of the most effective methods for image analysis since it extracts features in its convolutional layers and classifies the features in the fully connected layer. One-dimensional implementation of CNN (1D-CNN) has gained significant importance in extracting features in time series data and making robust predictions. This makes 1D-CNN a popular tool for analyzing sequential data, such as EEG signals^41^. We employed 1D-CNN to extract the characteristics of EEG signals and to predict emotions prior to the fuzzification.

### Fuzzy neural network (FNN)

The Fuzzy Neural Network, which merges the beneficial properties of fuzzy logic and neural networks, is an essential approach for intelligent information processing^42^. As a result, the fuzzy neural network technique has a powerful potential for both direct data processing through self-learning and efficient representation of structural knowledge. A multi-input fuzzy neural network system evaluates each input unit according to the degree to which it belongs to each fuzzy set.

The fuzzy rules used in the design of the FNN are represented using the “If–then” format, and they are as follows (Eq. 5):

5 $$P_{j} = IF\,u_{1}\,is\,P_{1j } \ldots \wedge u_{n}\,is\,P_{nj} , THEN\,y_{j} = w_{j}$$

where $P_{j}$ represents a fuzzy rule, $P_{{ij{ }}}$ represents fuzzy sets and w_j is a zero-order Takagi–Sugeno-Kang weight. The definition of the fuzzy set $P_{{ij{ }}}$, which uses a Gaussian membership function, is (Eq. 6):

6 $$P_{ij} = \exp \left\{ {\frac{{ - \left( {u_{1 - } m_{ij} } \right)}}{{2\sigma_{ij}^{2} }}} \right\}$$

where exp (.) is the exponential function and $m_{ij}$ and $\sigma_{ij}$ are the mean and standard deviation of a fuzzy set $P_{{ij{ }}}$, respectively.

## Proposed method

**Figure 2:**
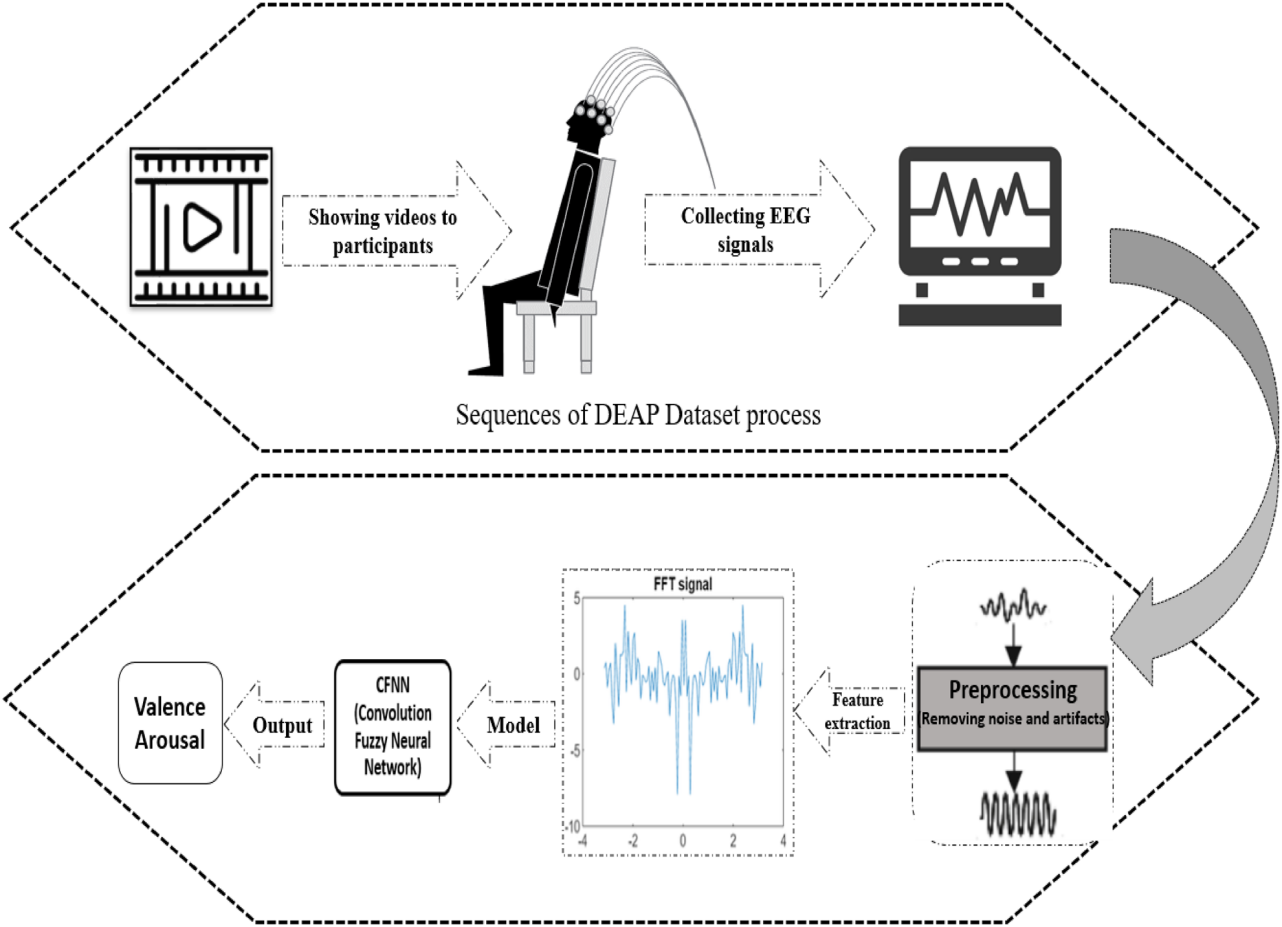
The proposed framework of emotion recognition in the current study.

The proposed method consisted of pre-processing to minimize noise and artifacts, feature extraction to obtain the most informative training data, and improving CFNN to classify emotions. Figure 2 demonstrates a brief description of the proposed methodology.

### Pre-processing and feature extraction

Even though the DEAP dataset was also provided as a pre-processed edition of its raw EEG data, it includes noise and several artifacts that can disrupt the analysis. In this study, EEG signals on 32 channels from 32 contributors viewing 40 videos in the DEAP dataset were used in the experiments, where EEG signals were first down-sampled to 128 Hz to collect accurate data content between 0 and 48 Hz^5^. EEG data were purged from the down-sampled data. The other elements in the signal, such as incremental waves, were also isolated from the analysis process after utilizing the bandpass filter.

One of the basic and challenging tasks in human emotion detection, which changes according to emotion fluctuation, is determining the appropriate features and attributes^8^. The performance of the emotion recognition model is strongly influenced by the quality of the features, which explains the importance of extraction features that are both strongly associated with emotion and have a good, accurate representation as the main component of emotion recognition^43^. By identifying the most valuable features for classification recognition from an enormous amount of feature data or generating a collection of "few but precise" characteristics with an extremely low probability of classification error, feature extraction is an approach that can decrease the dimensionality of feature space. Each feature derived from a signal provides specific details regarding the data and defines how signals behave. Feature extraction methods aim to propose a model with fewer features but more precision^44^. There are several techniques for feature extraction by EEG signals. There are three primary kinds of features: TDF (Time domain features), FDF (frequency domain features), and TFDF (time–frequency domain features)^45^.

A common signal processing technique used to convert time-domain signals to frequency-domain signals is Fourier analysis^45^. Fourier transformations are used in this study to break the EEG signal down into its frequency components. The FFT (Fast Fourier Transform) technique, which calculates a sequence's DFT (Discrete Fourier Transform), is a widely used method for computing the Fourier transform^46^. It yields the same results as evaluating the DFT definition explicitly, except that it is significantly faster. The formula of DFT is given in Eq. (7).

7 $$X_{k} = \mathop \sum \limits_{i = 0}^{N - 1} x_{i} \left( n \right)e^{{\frac{ - j2\pi ik}{N}}} \;\;\;for\; k = 0,1,2 \ldots N - 1$$

X_k denotes the discrete Fourier coefficient, N is the length of the accessible data, and x_i (n) is the input signal in the time domain^47^. The fraction of a signal's frequency bands that could not be confirmed in the time domain can be confirmed if the signal function is transformed into the frequency domain by Equation (FFT)^48^.

In this study, the FFT integrates information from the raw EEG database, considering the size of the window. All raw data from the DEAP dataset, specifically 40 EEG channels, is initially loaded for a single subject. Subsequently, the selected 14 channels of EEG data are presented collectively. Finally, the plotted representation of each of the 14 channels of EEG data is displayed as part of the third step. In step four, the FFT process demonstrates the conversion of each channel signal into a frequency domain using five power bands. In total, 14 channels were selected for this investigation, as shown in Table 1. The last stage demonstrates the combined frequency domain of the 14 channels. After the feature extraction process, the features were fed to the CFNN for classification.

**Table 1:** FFT description for parameters.

Selected channel		1, 2, 3, 4, 6, 11, 13, 17, 19, 20, 21, 25, 29, 31^23^
Bands	Theta	4–8 Hz
	Alpha	8–12 Hz
	Beta (lower frequency)	12–16 Hz
	Beta (higher frequency)	16–25 Hz
	Gamma	25–45 Hz

### The proposed convolutional fuzzy neural network model

The CFNN model is a neural network structure that integrates fuzzy logic and convolutional neural networks. It is primarily developed to deal with ambiguous or fuzzy data. Our proposed architecture combines the features extracted by the CNN with the fuzzy engine of the fuzzy neural network (FNN). The benefits of both network designs are combined in this approach.

The utilization of 1D-CNN has become increasingly significant in extracting features from time series data and generating reliable predictions. The filters in the convolution layers extract the features of 1D input sequences, and the most informative features are activated using the activation functions. The following layers provide more significant and distinguishable features corresponding to their labels in order to make proper predictions in the fully connected layers. However, it is challenging for 1D-CNN to optimize the extracted features due to the similarities or differences of time-series data. Even though 1D-CNN could achieve reasonable results, the improvement or the modifications of the extracted features with the fuzzification lead to an increased recognition ability of the models.

Fuzzification refers to the process of adding a fuzzy layer to the model. By using this layer, which transforms the input matrix (extracted features) into the fuzzy domain, high-dimension feature extraction can be accomplished using a convoluted representation of the result with the ability to handle noise in data. The fuzzy set’s estimate is carried out following Eqs. (8) and (9), which show the probabilities of the components existing in the domain of fuzzy numbers^49^.

8 $$\hat{X}\, = \,Fuzzification\left( {X_{i,j } | cx_{i,j} } \right)$$

9 $$x_{i,j} = possibility {(}x_{i,j} {|}MF_{i,j} ) = \max MF_{i,j} \delta \left( {x - x_{i,j} } \right)$$

Here i and j represent the element x’s index in the input matrix X, and cx is the center of the input fuzzy membership function. $\delta \left( {x - x_{i,j} } \right)$ represents the Kronecker delta function.

In the proposed model, the CNN architecture includes two 1D convolution layers. In the first convolution layer, we aimed to increase the dimension of features using 64 filters with a 5 × 5 kernel, and in the second convolutional layer, we compressed the feature map by 32 filters with 3 × 3. In order to minimize the computational cost and the dimension of the feature map, max-pooling was applied, ensuring the choice of pertinent features. The extracted features are flattened and then fed to the fuzzification layer to eliminate noise in the data.

After the fuzzification of the features, batch normalization is applied to avoid over-fitting, and a dense layer with two nodes is used prior to the defuzzification to improve the classification performance.

Defuzzification is the process of reversing fuzzified values into the crisp values where the noise-free and more informative features are transmitted to the final output layer. The defuzzification procedure calculates the crisp value, ν_i, using Eq. (10).

10 $$\nu_{i} = defuzzy \left( {x_{i} } \right) = \frac{{\sum C_{y } x_{i} }}{{\sum x_{i} }}$$

where $C_{{y{ }}}$ represents the defuzzification membership function's core. The weight assigned to a fully connected layer is denoted as $W_{{fc{ }}}$.

**Figure 3:**
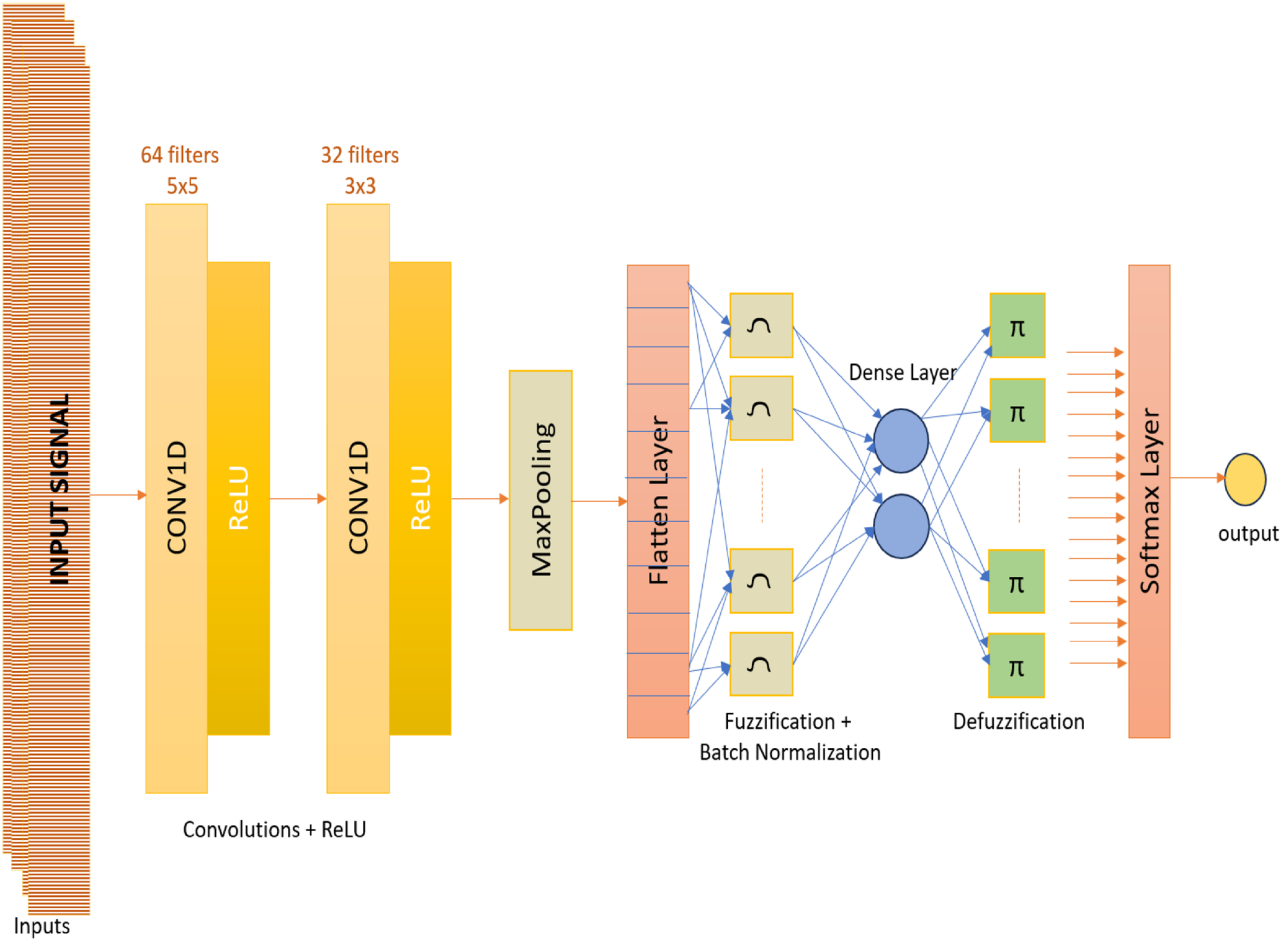
Structure of the proposed convolutional fuzzy neural network.

Figure 3 demonstrates the general structure of the proposed CFNN model, and Table 2 presents detailed information about the parameters and layers of the proposed model.

**Table 2:** Detailed information about the parameters and layers of the proposed model.

No	Layer	Filter/Kernel/Node	Output shape	Param#
1	Conv1D	64/5 × 5/–	(None, 73, 1)	384
2	Conv1D	32/3 × 3/–	(None, 73, 64)	6176
3	Max pooling	–/–/–	(None, 73, 32)	0
4	Flatten	–/–/–	(None, 1152)	0
5	Fuzzy	–/–/–	(None, 2)	4608
6	Batch normalization	–/–/–	(None, 2)	8
7	Dense	2	(None, 2)	6
8	Defuzzy	–/–/–	(None, 2)	4
9	Activation	–/–/–	(None, 2)	0
Total parameters: 11,186				
Trainable parameters 11,182				
Non-trainable parameters: 4				

## Results

### Experiments

Several experiments, which are varied from the training and testing split ratio to feature extraction window size, have been performed to analyze the proposed model's recognition ability and conduct comparative studies with state-of-the-art methods. Multiple experiments have been conducted, ranging from adjusting the training and testing split ratio to varying the size of the feature extraction window, as well as testing with different percentages of training and testing data. These experiments are conducted to analyze the recognition capability of the proposed model and compare its performance with state-of-the-art methods. The experiments are performed on an Intel (R) Core (TM) i5-5200U CPU @ 2.20GHZ, 64 GB of RAM, and NVIDIA GeForce 840 M PC. The proposed model was implemented in Google Collaboratory, a hosted Jupyter Notebook service. The Python version is 3.10.12, and the Tensorflow version is 2.12.0.

In addition to the proposed improved CFNN method, we trained SVM, KNN, 1D-CNN, and Bi-LSTM for the binary classification of emotions (arousal and valence) to provide a comparative study. All models were trained using 75% of data obtained from the feature extraction process as training data and 25% as test data. The proposed model's learning rate and batch size were set to 0.01 and 256, respectively, and the Adam optimizer was used. The training was stopped after 100 epochs.

The window size of FFT was varied between 4 and 128, doubled at each experiment, and the training and testing ratio was set to 90–10%, 80–20%, and 75–25%. In addition to the hold-out experiments, a five-fold cross-validation experiment was performed on the proposed model to obtain consistent results.

### Results

When the proposed model was trained using different training and testing ratios, the highest accuracy, F1-score, and precision were obtained in five-fold cross-validation experiments (K = 5), where all data were considered in separate training and testing folds. The highest recall and second-best scores of other metrics were achieved in a 75:25 training and testing ratio. The other hold-out ratios could not outperform the scores obtained in the K = 5. Based on the obtained results, to minimize training time and speed up the analyses, the rest of the experiments are performed using a 75:25 hold-out ratio. Table 3 presents the results obtained in the hold-out and K = 5 experiments.

**Table 3:** Performance of the proposed CFNN using varying train/test splits.

Train/Test	Results			
	Accuracy*	Precision	F1-score	Recall
90:10	96.94% (96.86–97.01)	95.93%	96.43%	96.93%
80:20	96.85% (96.78–96.92)	96.25%	96.49%	96.73%
75:25	*97.24%* *(97.18–97.30)*	*96.47%*	*96.79%*	**97.12%**
K = 5	**97.79% (97.71–97.87)**	**97.70%**	**97.67%**	*96.98%*

*Values in parentheses indicate a 95% confidence interval; bold values indicate the best values, while italic values indicate the second highest.

Fluctuated recognition rates were obtained when the proposed model was trained with different window sizes (4, 8, 16, 32, 64, 128). It made the task challenging to conclude which window size was more effective and informative; however, it should be noted that 4 and 8 window sizes might not be suitable for classifying emotions. Table 4 presents the results obtained by the proposed method with different window sizes.

**Table 4:** Performance of the proposed CFNN using different window sizes.

Window size	Accuracy*	Precision	F1-score	Recall
4	97.06% (96.98–97.14)	96.50%	96.58%	96.67%
8	97.24% (97.17–97.31)	96.47%	96.79%	97.12%
16	97.36% (97.29–97.43)	94.85%	96.87%	**98.98%**
32	97.88% (97.83–97.93)	**99.12%**	*98.15%*	97.19%
64	*98.54%* *(98.49–98.59)*	96.47%	96.79%	97.12%
128	**98.70%** **(98.66–98.74)**	*99.01%*	**98.49%**	*97.98%*

*Values in parentheses indicate a 95% confidence interval; bold values indicate the best values, while italic values indicate the second highest.

Based on the hold-out and window size experiments, the proposed model with a 75:25 training and testing ratio and 32 window size achieved 98.39 and 97.93 F1 scores for valence and arousal, respectively. The proposed CFNN model resulted in enhanced classification capabilities, yielding an average accuracy of 98% when employing fusion techniques. This improved performance can be attributed to fuzzy logic’s capacity to emulate human reasoning. As the weights were modified periodically, it caused a reduction in overfitting and gave the best outcomes. Table 5 shows the accuracy, precision, recall, and F1-score results of the proposed model in valence and arousal on the DEAP dataset.

**Table 5:** Results of the proposed CFNN method on the DEAP dataset (in 75–25%).

Model	Emotion	Precision	Recall	F1-Score	Accuracy*
CFNN	Valence	98.57	98.21	98.39	98.21 (98.16–98.26)
	Arousal	96.78	99.11	97.93	98.08 (98.01–98.15)

*Values in parentheses indicate a 95% confidence interval.

**Figure 4:**
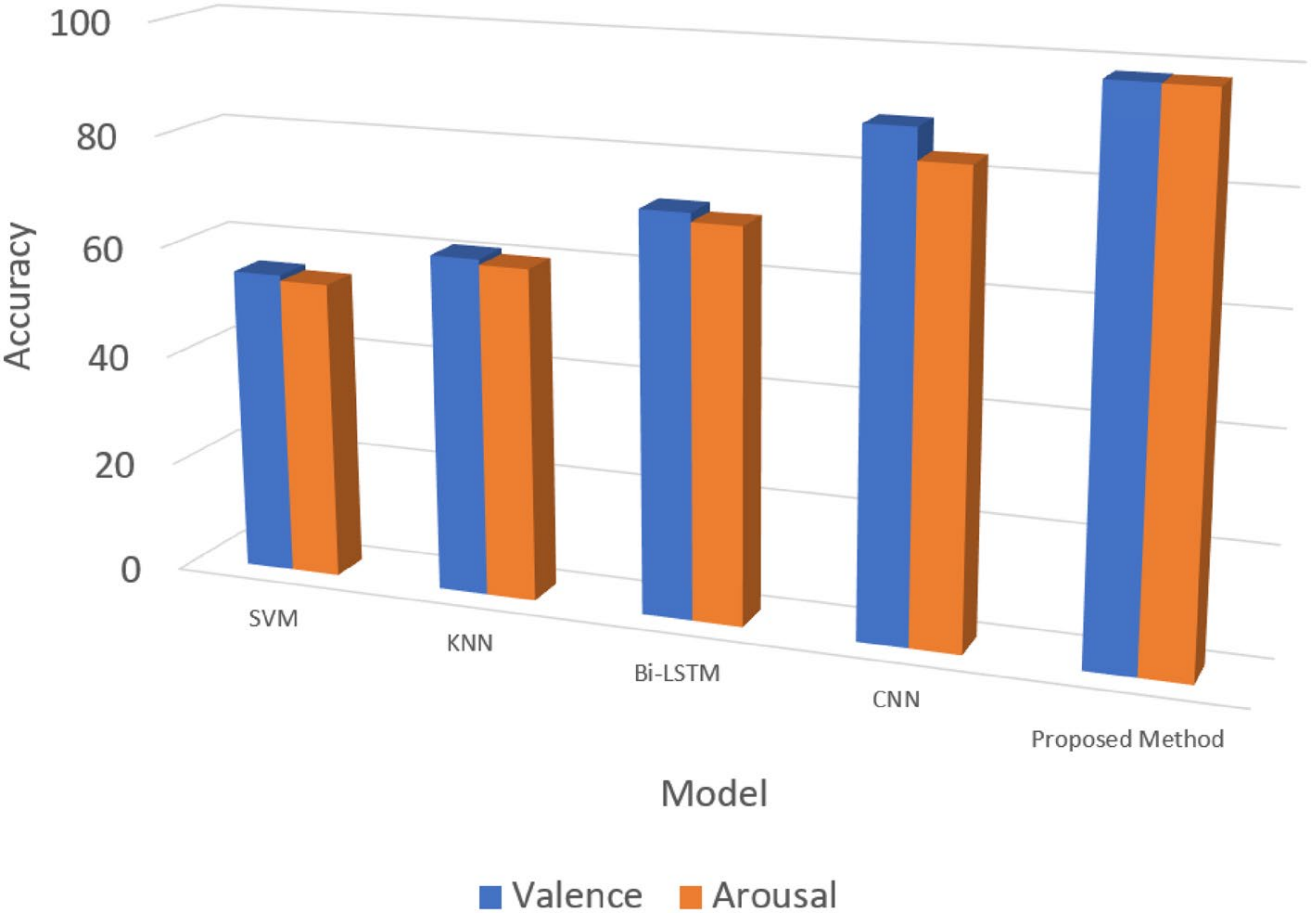
Graphical visualization of comparison results.

When other comparative methods were considered, it was clear that the SVM and KNN failed to produce correct emotional classifications using the extracted features. The SVM obtained 55% and 54% accuracy for valence and arousal, while the KNN achieved 61.00% and 60.00%, respectively. Even though the Bidirectional-LSTM improved the recognition rates by achieving 72.01% for valence and 70.42 for arousal, it could not outperform 1D-CNN. Among those methods, 1D-CNN achieved superior results (88.87% for valence and 83.35% for arousal); however, the optimal results were obtained by the proposed method by 98.21% and 98.08%, respectively. Table 6 presents the results obtained in the comparative study with the state-of-the-art methods, and Fig. 4 visualizes the results.

**Table 6:** Comparison results of the proposed CFNN method with state-of-the-art methods.

Accuracy (%)		
Model	Valence	Arousal
SVM	55.00	54.00
KNN	61.00	60.00
Bi-LSTM	72.01	70.42
CNN	88.87	83.35
Propose-Method	98.21	98.08

### Limitations

Emotional recognition studies have different challenges, such as data acquisition procedures, tools, aims, etc. These differences create varied and different datasets, which prevent the use of external datasets to validate the proposed methods. The proposed model has not been implemented for different datasets that have different characteristics, and the generalization of the results in real-time applications requires further investigation.

## Discussion

Recent and the most accurate studies are focused on the deep learning approach; however, the pre-processing or relevant approaches were also effective on the recognition ability of the models. In the study by Al-Nafjan et al.^50^, PSD was used for feature extraction and DNN for classification. They achieved 82% in both valence and arousal accuracy. Alhagry et al.^51^ utilized LSTM to learn features from the signals. They achieved 85.45% and 85.65% for valence and arousal. However, Xing et al.^24^ employed SAE (Stack AutoEncoder) with LSTM-RNN to fix the linear EEG signals problem. The accuracies obtained were 81.10% and 74.38% in valence and arousal, respectively. Furthermore, Iyer et al.^39^ implemented a CNN and LSTM-based hybrid model in which DE was used as feature extraction, and they obtained 65% accuracy. In addition, Sharma et al.^52^ considered valence and arousal emotion for classification, and PSD was used for feature extraction. They applied CNN and LSTM models, which, as the results obtained, were 85.23%, 86.50 for CNN, and 87.68%, 87.98% for LSTM on the valence and arousal. In one of the most influencing studies, Singh et al.^53^ developed a CNN 1D and Bi-LSTM model as classification and achieved 92.29% and 90.33% in two classes. Another influencing accurate was performed by Yang et al.^54^ by suggesting a multi-column structure to enhance the accuracy of the CNN-based model. They achieved 90.01% in valance and 90.65% in arousal.

However, the complexity of signals and the variety of the pre-processing approaches and classifiers produce fluctuated results in emotion recognition. In our study, converting the extracted features of the flattened layer into fuzzy values with learnable parameters and applying defuzzification provided the representation of the features more informative. This improved the recognition ability of the model, and the proposed method produced superior results to the recent studies by 6–17%. Table 7 compares the proposed method with the recent studies for the same dataset.

**Table 7:** Comparison of the proposed method with the recent studies for the DEAP dataset.

Study	Feature extraction	Classifier	Dataset	Results	
				Valence	Arousal
^50^	PSD, Asymmetry features	DNN	DEAP	82.00	82.00
^51^	–	LSTM	DEAP	85.45	85.65
^27^	Signal framing, Frequency band power, Pearson correlation	SAE + LSTM	DEAP	81.10	74.38
^39^	Differential entropy	CNN + LSTM	DEAP-SEED	65 (DEAP)	
^52^	PSD	CNN	DEAP-SEED	85.23	86.50
^52^	PSD	LSTM	DEAP-SEED	87.68	87.98
^53^	–	1DCNN + LSTM	DEAP	92.29	90.33
^54^	–	CNN	DEAP	90.01	90.65
^55^	–	LSTM-Attention	DEAP	90.10	83.30
Proposed	FFT	CFNN	DEAP	98.21	98.08

## Conclusion

The rate of false positives and the possibility of image manipulation in image processing can affect the accuracy of results. However, such manipulation cannot occur in EEG brain wave signals as it cannot be tampered with. For this purpose, recording brain signals makes recognizing and investigating emotions easier. This study aimed to improve the accuracy of emotions obtained from EEG signals using deep learning and fuzzy logic. EEG signals were pre-processed to eliminate noise, features were extracted in the frequency domain using FFT, and the classification was performed using the improved CFNN model.

A comparative study was performed using four trained state-of-the-art methods, and the results suggested that the proposed method outperformed other methods in all metrics. Additionally, the proposed model was compared with the recent studies, and the proposed method achieved superior results for the same dataset. The performance of the proposed method was recorded as 98.21% and 98.08% for valence and arousal. The performance of the proposed method suggests that the conversion and representation of the extracted features as fuzzy posterior to the convolutional layers provide more informative features in the recognition phase. Our future work will include the multiclass implementation of the proposed method.

## Data Availability

The dataset used in this research can be accessed at: https://www.eecs.qmul.ac.uk/mmv/datasets/deap/ (accessed on 13 February2022.
